# Laser speckle contrast imaging for real-time quantification of colonic perfusion following high versus low tie in anterior rectal resection surgery: a pilot prospective study

**DOI:** 10.1097/JS9.0000000000003461

**Published:** 2025-09-12

**Authors:** Jia-Zi Yu, Tao Zhou, Yu-Peng Zheng, Mian Yang, Bo Zhou, Jia-Ze Sun, Jin-Ling Lu, Wei Cui, Peng-Cheng Li

**Affiliations:** a Department of Colorectal and Anal Surgery, Ningbo Medical Treatment Center, Li Huili Hospital, Ningbo, People’s Republic of China; b Department of Colorectal and Anal Surgery, Li Huili Hospital of Ningbo University, Ningbo, People’s Republic of China; c Britton Chance Center of Biomedical Photonics, Advanced Biomedical Imaging Facility, Wuhan National Laboratory for Optoelectronics, Huazhong University of Science and Technology, Wuhan, Hubei, People’s Republic of China; d State Key Laboratory of Digital Medical Engineering, Key Laboratory of Biomedical Engineering of Hainan Province, School of Biomedical Engineering, Hainan University, Sanya, People’s Republic of China

**Keywords:** anastomosis leakage, laser speckle contrast imaging (LSCI), perfusion, rectal cancer

## Abstract

**Background:**

The optimal management of the inferior mesenteric artery (IMA) in rectal cancer surgery remains controversial owing to its unclear impact on bowel perfusion. This study aimed to objectively evaluate perfusion differences between high tie (HT) and low tie (LT) using laser speckle contrast imaging (LSCI) and determine its value in guiding surgical decisions.

**Methods:**

Patients who underwent laparoscopic anterior rectal resection for rectal or rectosigmoid cancer were prospectively enrolled for either HT or LT. The primary outcome was the maximum perfusion distance (MPD). The secondary outcomes included the Speckle Flow Index (SFI) at the transection site and the frequency of LSCI-guided surgical revisions.

**Results:**

After propensity score matching (*n* = 30/group), no significant overall difference was found in the median MPD (*P* = 0.12) or mean SFI (*P* = 0.20) between the HT and LT groups. However, a key finding was the identification of a high-risk HT patient subgroup with critically short MPD, a phenotype that was absent in the LT cohort. Consequently, LSCI guidance prompted surgical revision in 16.7% of the HT patients (vs. 0% in the LT group). Ultimately, this individualized approach resulted in an equally low anastomotic leakage rate (3.3%) in both the cohorts.

**Conclusion:**

Our results from this pilot study are hypothesis-generating. While the average perfusion did not differ significantly between IMA management techniques, HT posed a unique risk by creating a patient subset with critically compromised perfusion. Real-time LSCI assessment proved effective in identifying these high-risk individuals intraoperatively, prompting timely surgical revisions and thereby reducing the incidence of anastomotic leakage.

**HIGHLIGHT**

- Based on laser speckle contrast imaging (LSCI) visualization of bowel perfusion, we introduce a novel metric, the maximum perfusion distance, for objective assessment of segmental bowel perfusion.
- High-tie, not low-tie, ligation introduces a unique risk, creating a subset of patients with critically compromised bowel perfusion.
- Real-time LSCI robustly identifies these high-risk cases and guides corrective surgical revisions.
- LSCI-guided intervention enables safe anastomotic outcomes regardless of the chosen ligation technique.

## Introduction

Despite standardized surgical protocols for anterior resection (ARR) in curative intent rectal cancer surgery^[1–4]^, there remains an ongoing debate regarding the management of the inferior mesenteric artery (IMA), specifically, the choice between high tie (HT) at the origin of the IMA and low tie (LT) that preserves the left colic artery (LCA)^[5]^. This debate remains unresolved because of the absence of objective tools to demonstrate whether LCA preservation enhances bowel blood perfusion and improves anastomotic outcomes.

Intraoperative perfusion assessment technologies aim to resolve this uncertainty. While indocyanine green fluorescence angiography (ICG-FA) is increasingly used, its reliance on subjective visual interpretation and single-use limitation hinders objective comparison and dynamic assessment^[6,7]^. Laser speckle contrast imaging (LSCI) emerges as a superior alternative, offering real-time, objective, and repeatable quantitative data without the need for fluorescent dye^[8]^. Despite its clear advantages, the potential of LSCI to systematically compare perfusion outcomes of HT versus LT remains unexplored. Furthermore, a robust, clinically relevant metric to assess perfusion over a segment of bowel, rather than at a single point, has been lacking.

Therefore, this study aimed to objectively compare bowel perfusion after HT and LT using LSCI. To achieve this, we introduce a novel primary metric: the maximal perfusion distance (MPD), which quantifies the length of viable proximal bowel available for a tension-free anastomosis. In addition, we measured the perfusion intensity at the proximal transection site using the Speckle Flow Index (SFI) and evaluated the frequency of LSCI-guided revisions to the initial surgical plan in each group. We hypothesized that the LT approach would result in a significantly longer MPD compared to the HT approach. By providing the first objective data on these critical parameters, this work aims to resolve a long-standing surgical dilemma and lay the foundation for personalized, evidence-based management of the IMA in rectal cancer surgery.

## Methods

### Patient selection

**Figure 1.**
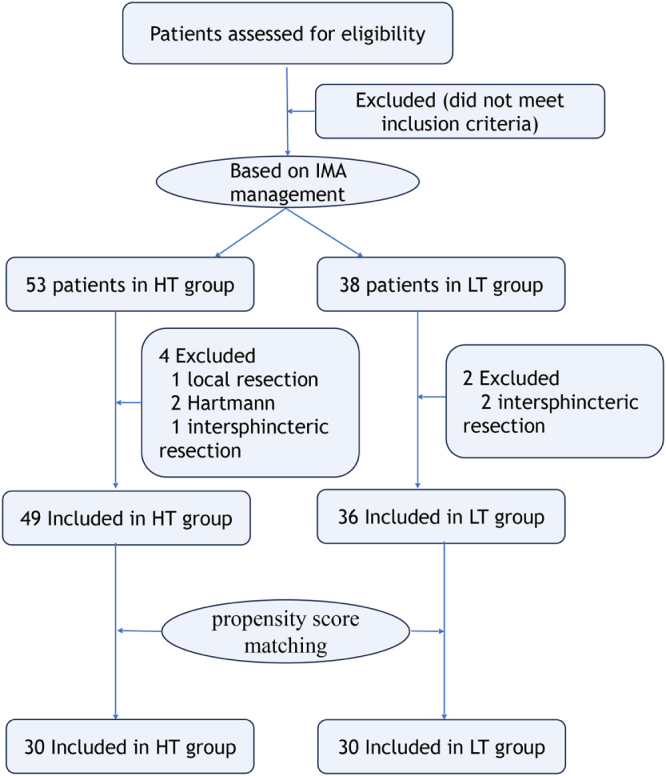
Study flowchart.

This single-center, prospective observational study was conducted at a district general hospital and a teaching hospital. This study adhered to the STROCSS guideline recommended for observational studies^[9]^. Patients diagnosed with rectal or rectosigmoid cancer between May and October 2024 were enrolled in the study. The inclusion criteria were as follows: (1) confirmed diagnosis based on pathological reports, (2) patients who had not received any prior treatment, and (3) patients aged ≥18 years. The exclusion criteria were as follows: (1) a history of previous abdominal surgery or neoadjuvant chemotherapy/radiation affecting bowel perfusion or anastomosis; (2) patients requiring emergency surgery due to acute complications; and (3) intraoperative findings necessitating a shift to alternative procedures such as local excision, abdominoperineal resection, Hartmann’s operation, or intersphincteric resection. The patient enrollment flowchart is shown in Figure 1. The study protocol adhered to the principles of the Declaration of Helsinki and was approved by the local ethics committee (Approval No. KY2025SL104-01).

### Intraoperative bowel perfusion assessment

To minimize subjective bias and ensure consistency in the application of LSCI-guided surgical decisions, all participating surgeons completed a standardized training protocol before the study commenced, as detailed in Supplemental Digital Content Methods, available at: http://links.lww.com/JS9/F110. A video walkthrough of the LSCI acquisition workflow is provided as a Supplementary Video (also available at: http://links.lww.com/JS9/F240.

At the beginning of a laparoscopic-assisted LAR procedure, the surgeon first identified and marked the starting point of the sigmoid colon, as depicted in Supplemental Digital Content Methods, available at: http://links.lww.com/JS9/F110 and Supplemental Digital Content Figure 1, available at: http://links.lww.com/JS9/F110. Following the completion of laparoscopic rectal transection, a small abdominal wall incision was created, and a wound protector was inserted. The proximal colon was exteriorized through the incision. After ensuring that the colon is adequately flattened, blood perfusion measurements can begin. The perfusion assessment was performed using an LSCI system (SIM BFI WFTIM; SIM Opto-Technology Co., Wuhan, China)^[9]^. The monitor detector was positioned 30–40 cm above the specimen, offering a maximum field of view of 14.8 × 14.8 cm to ensure adequate illumination of the entire specimen area, as shown in Supplemental Digital Content Figure 2C, available at: http://links.lww.com/JS9/F110. Real-time speckle patterns were captured at 1 Hz (512 × 512 pixels) and processed using dedicated software (SIM BFI v2.0) to create 2D perfusion maps. Bowel perfusion was assessed at two standardized time points for each surgical procedure, as detailed in the Supplemental Digital Content Methods, available at: http://links.lww.com/JS9/F110 and Supplemental Digital Content Figure 4A, B, available at: http://links.lww.com/JS9/F110. Following LSCI-guided selection of the proximal bowel transection line, the anvil head of the circular stapler was inserted. The bowel was then repositioned into the abdominal cavity and the anastomosis was completed laparoscopically. Finally, ICG fluorescence was employed to confirm perfusion at the anastomosis site, as shown in Supplemental Digital Content Figure 4C, available at: http://links.lww.com/JS9/F110.

### Outcome measurements

The primary measurement was the MPD of the proximal bowel, determined by measuring the distance from the ischemic line of demarcation (LOD) to the starting point of the sigmoid colon (Supplemental Digital Content Fig. 5A–C, available at: http://links.lww.com/JS9/F110), as detailed in Supplemental Digital Content Methods. A secondary measurement involved assessing changes in transection line decisions following LSCI-based bowel perfusion visualization, along with *post hoc* quantification of perfusion at the proximal anastomosis site, as detailed in Supplemental Digital Content Methods, available at: http://links.lww.com/JS9/F110 and Supplemental Digital Content Figure 5E–G, available at: http://links.lww.com/JS9/F110.

### Patients followed up

All patients were followed up for a period of 90 days postoperatively to assess surgical and oncological outcomes. Follow-up was conducted via outpatient visits or telephone calls. Patients diagnosed with an anastomotic leak (AL) prior to discharge were monitored through outpatient clinic visits. The primary outcomes evaluated during this period were the incidence of clinical and subclinical AL and the patient’s oncological status. AL was defined and graded in accordance with the criteria detailed in the Supplemental Digital Content Methods, available at: http://links.lww.com/JS9/F110. Due to the inherent challenges in diagnosing subclinical AL in patients with a protective terminal ileostomy, these individuals underwent an additional endoscopic examination at 90 days postoperatively to ascertain the integrity of the anastomosis. Oncological follow-up was performed in accordance with the National Comprehensive Cancer Network (NCCN) Guidelines^[10]^ to monitor for local recurrence and distant metastasis. This surveillance involved comparing postoperative blood tumor markers with preoperative baseline levels, and conducting imaging studies such as abdominal ultrasound, enhanced CT, or MRI as clinically indicated.

### Statistical analysis

All data were analyzed using SPSS statistical software (version 22.0; SPSS Inc., Chicago, IL, USA) and R software environment, version 3.5.3 (R Foundation for Statistical Computing). Descriptive statistics were calculated for all relevant variables, including the mean, standard deviation (SD), and frequency. The differences between the groups were assessed using the test or chi-squared (*χ*^2^) test, as appropriate. The differences between groups were assessed using either a *t*-test or a *χ*^2^ test, as appropriate. All tests were two-sided, and the significance level was set at *P* < 0.05.

## Results

### Baseline characteristics

This study enrolled 91 consecutive patients, with 53 assigned to the HT group and 38 to the LT group, between June and October 2024. The patient characteristics are shown in Supplemental Digital Content Table 1, available at: http://links.lww.com/JS9/F110. After propensity score matching in a 1:1 ratio, 30 patients were included in each group. The HT group consisted of 19 men and 11 women, with a mean (SD) age of 57.8 (10.7) years, while the LT group comprised 17 men and 13 women, with a mean (SD) age of 60.1 (9.1) years (Table 1). The baseline demographic characteristics and clinicopathological features were generally balanced and comparable between the two groups.

**Table 1:** Basic characteristics of patients in the HT and LT groups

Variables	HT (30)	LT (30)	Statistics value	*P*-value
Sex				
Male	19	18	0.07	0.79
Female	11	12		
Age				
≥60	22	23	0.09	0.77
<60	8	7		
BMI				
≥25	7	10	0.74	0.39
<25	23	20		
Blood pressure				
Normal	21	18	0.66	0.42
High	9	12		
Diabetes				
Yes	3	3	0.00	1.00
No	27	27		
Ventilatory impairment				
Yes	3	3	0.00	1.00
No	27	27		
Cardiovascular comorbidity				
Yes	4	4	0.00	1.00
No	26	26		
Atherosclerosis				
Yes	7	9	0.34	0.56
No	23	21		
Tumor location				
Upper	26	24	0.48	0.49
Mid and distal	4	6		
Tumor stage				
I	2	6	3.10	0.38
II	13	9		
III	13	14		
IV	2	1		
LCA to the root of the IMA (cm)	40.0 ± 15.1	38.5 ± 14.4	0.40	0.70
LCA diameter (mm)	2.1 ± 0.6	2.1 ± 0.8	0.18	0.86
LCA type				
I	20	17	1.08	0.78
II	6	6		
III	3	5		
IV	1	8		

BMI: body mass index; HT: high tie; IMA: inferior mesenteric artery; LCA: left colic artery; LT: low tie.

### Effect of LSCI on the assessment of intraoperative bowel blood perfusion

The LSCI successfully provided visualized perfusion assessments for all participants. Typical LSCI images displayed three distinct blood perfusion areas: adequate perfusion (red), indicating healthy, well-vascularized tissue; borderline perfusion (yellow/green), indicating potentially compromised but possibly viable tissue; and critically compromised perfusion (blue), indicating ischemic tissue unsuitable for anastomosis (Supplemental Digital Content, Fig. 2B, available at: http://links.lww.com/JS9/F110). The boundary between adequate perfusion and borderline perfusion regions was defined as LOD (Supplemental Digital Content, Fig. 2A, available at: http://links.lww.com/JS9/F110). From a pre-study pilot experiment with 10 patients used to train surgeons, SFI values were found to differ significantly among adequate, borderline, and compromised perfusion zones (*P* < 0.01, Supplemental Digital Content, Fig. 2D, available at: http://links.lww.com/JS9/F110). When surgeons determined the transection line and ligated the marginal vascular arch, the LOD was aligned exactly with the ligation point, as illustrated in Supplemental Digital Content Figure 3D, available at: http://links.lww.com/JS9/F110.

### Results of proximal bowel blood perfusion: HT versus LT approaches

**Figure 2.**
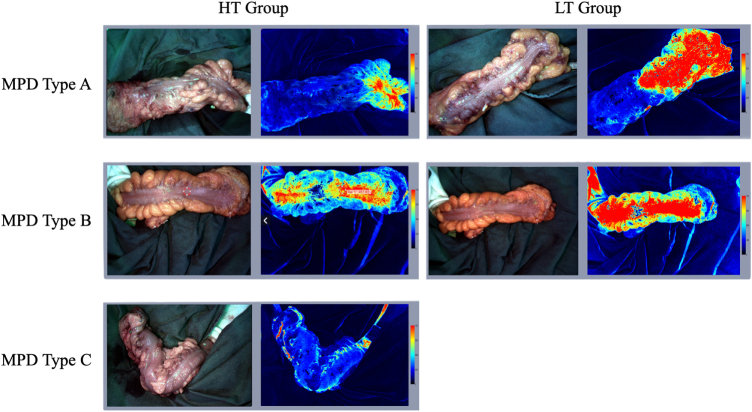
Intraoperative LSCI revealed distinct phenotypes of bowel perfusion, providing a hypothesis-generating framework for understanding inter-patient variability. (A) The short MPD phenotype, characterized by markedly reduced perfusion; (B) the typical MPD phenotype, representing the predominant perfusion pattern observed; and (C) the long MPD phenotype, characterized by significantly extended perfusion.

**Figure 3.**
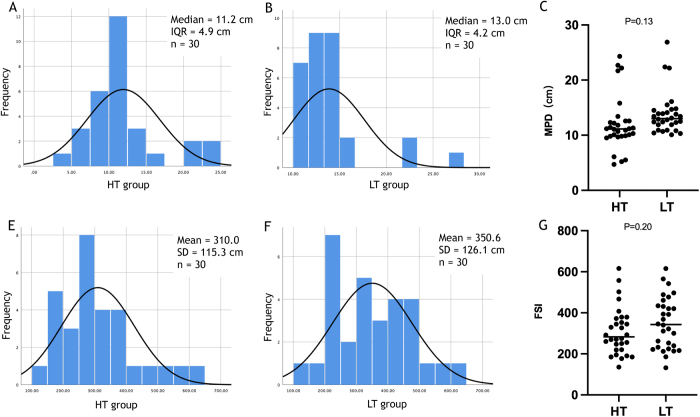
The results of MPD and SFI values for the HT and LT groups based on LSCI. (A–B) Histograms of MPD (cm) for the HT group (A) and LT group (B) measured by LSCI; annotated medians and interquartile ranges (IQRs) are shown (n=30 per group).(C) Comparison of individual MPD values between the HT and LT groups.; two-sided between-group comparison P=0.13. (E–F) Histograms of SFI values for the HT group (E) and LT group (F); annotated means and standard deviations (SDs) are shown (n=30 per group).(G) Comparison of individual SFI values between the HT and LT groups; P=0.20.

The MPD results in both the groups showed a non-normal distribution. The median MPD in the HT group was 11.2 cm (range: 4.7–23.9 cm; IQR: 4.9 cm), whereas in the LT group, the median MPD was 13.0 cm (range: 4.3–26.9 cm; IQR: 4.2 cm). However, this difference was not statistically significant between the two groups (*P* = 0.12; Fig. 3A–C). Seven (11.7%) demonstrated a significantly prolonged MPD, with three patients in the LT group and four patients in the HT group. In contrast, within the HT group, four patients (13.3%) had critically poor perfusion (exceptionally short MPD), as depicted in Figure 2A, while no such occurrences were observed in the LT group. SFI values at the proximal anastomosis site followed a normal distribution. The mean (SD) SFI value in the HT group was 310.0 (115.3), while in the LT group, the mean (SD) SFI value was 350.6 (126.1). This difference was not statistically significant between the two groups (*P* = 0.20; Fig. 3E–G).

LSCI influenced intraoperative surgical transection line decision-making in five cases (10.0%) among the enrolled 60 patients, all of which occurred in the HT group, accounting for 16.7% (5 out of 30). In the HT group, the initial transection line was revised in four patients because it was located distal to the LOD, as determined by LSCI. These four patients also had a significantly short MPD. Additionally, in one patient, although the transection line was located within the area of normal perfusion, it was shifted distally to ensure tension-free anastomosis.

### Correlation between marginal artery anatomy and perfusion phenotypes

Preoperative CTA reconstructions revealed different anastomotic patterns of the marginal artery arch at the splenic flexure. Among them, 7 patients were suspected to have an accessory middle colic artery (AMCA); in 36 patients, a bypass connection was found between the left branch of the MCA and the ascending branch of the LCA; in 5 patients, the ascending branch of the LCA connected directly to the marginal artery of the transverse colon; and in 12 patients, the LCA connected to the marginal artery of the descending colon.

Interestingly, all 7 patients who exhibited a significantly prolonged MPD were observed to have an AMCA on CTA. In contrast, the four patients in the HT group with an exceptionally short MPD shared a different anatomical characteristic. Although their CTA scans showed no direct evidence of a focal “weak point” in the marginal artery, such as Griffith’s point^[11]^, the key finding was the absence of a bypass connection between the ascending branch of the LCA and the left branch of the MCA.

### Operative and postoperative outcomes

All patients were followed for at least 90 postoperative days. No intraoperative complications occurred in either of the groups (Table 2). The operation time was 167.3 ± 64.9 min in the HT group and 183.7 ± 77.1 min in the LT group. Postoperatively, no significant differences were identified in the rate of postoperative complications (Table 2). None of the patients in either group developed mortality. Only one Grade A AL occurred in each group. There were no significant differences in the length of postoperative hospital stay between the two groups.

**Table 2:** Operative and postoperative outcomes in the HT and LT groups

Variables	HT (30)	LT (30)	Statistics value	*P*-value
ASA score				
I	2	2	0.00	1.00
II	31	31		
III	4	4		
Operating time (min)	167.3 ± 64.9	183.7 ± 77.1	0.89	0.38
Blood loss (ml)	20.3 ± 5.6	28.2 ± 6.1	1.26	0.21
Harvested lymph nodes (*n*)	18.2 ± 6.1	17.6 ± 7.8	0.33	0.74
Complication rate (%)	7 (23.3)	6 (20)	0.10	0.75
Wound infection	3 (10)	3 (10)	0.00	1.00
Ileus	2	1	0.35	0.55
Urinary Retention	1	2	0.35	0.55
Pulmonary complications	1	0	1.02	0.31
Anastomotic leaks	1	1	0.00	1.00
Hospital stay (days)	7.5 ± 2.9	6.9 ± 3.2	0.76	0.45

ASA: American Society of Anesthesiologists; HT: high tie; LT: low tie.

## Discussion

LSCI is increasingly recognized as a valuable non-invasive tool for the real-time, objective assessment of tissue perfusion^[13–15]^. Consistent with previous reports^[16]^, our study confirmed LSCI’s ability to accurately reflect bowel perfusion dynamics; the observed shift in the LOD precisely corresponded to the ligation site of the marginal vascular arch (Fig. 2), validating its utility in this surgical context.

To our knowledge, this is the first study to introduce MPD, as quantified by LSCI, as a primary metric for evaluating bowel perfusion in ARR surgery. The development of this objective, large-scale assessment was driven by the historical limitations of methods that could not provide a similarly accurate and visualized assessment of perfusion over an extensive segment of the bowel. The clinical utility of MPD is of clear clinical importance: it defines the extent of well-perfused bowel available for selection, which is fundamental for constructing a safe and tension-free anastomosis^[17,18]^. Compared to the study by Han *et al*^[19]^, which utilized ICG-FA for quantitative evaluation of colonic perfusion following high versus low tie techniques, LSCI provides greater objectivity and operational flexibility. Unlike ICG-FA, it is not influenced by dye washout or residual fluorescence, and allows for real-time, repeatable measurements without the need for contrast reinjection. This unique capability enables the functional quantification of perfusion parameters such as MPD. Building on this advantage, our team is launching a prospective clinical trial in which temporary occlusion of the LCA will be used to dynamically evaluate its impact on MPD. This approach may offer new insights into collateral perfusion capacity and inform more individualized surgical planning in colorectal procedures.

The study found no significant difference in MPD when comparing the HT group (11.2 cm, IQR: 4.9 cm) with the LT group (13.0 cm, IQR: 4.2 cm). The resultant 2.0-cm longer median MPD associated with the LT approach, while not statistically significant (*P* = 0.12), is also unlikely to be clinically sufficient to substantially alter anastomotic reconstruction options in most instances. This observation may contribute to understanding why large randomized controlled trials have often failed to demonstrate a significant reduction in AL risk with the LT technique^[20]^. While strict anatomical landmarks were established to standardize MPD measurement, we acknowledge the potential for minor operator-dependent variations. However, this is considered of secondary importance relative to the principal finding of our investigation. Indeed, although MPD was the primary outcome, the most notable finding was not merely the modest difference in MPD between the two IMA management techniques. Rather, it was the emergence of three distinct perfusion patterns based on MPD measurements, as illustrated in Figure 2. Among these, two contrasting phenotypes held particular clinical significance. A subset of patients in both the HT and LT groups demonstrated exceptional perfusion, characterized by remarkably long MPDs indicative of virtually no ischemic bowel. Conversely, a subgroup of patients exhibiting critically short MPDs, and consequently extensive ischemia, was observed within the HT group. This highlights that the HT technique, while potentially adequate on average, carries a demonstrable risk of severely compromising perfusion length in certain individuals. The stark dichotomy in perfusion outcomes between the two groups cannot be attributed to the anatomical characteristics of the LCA. Our analysis confirmed that key LCA features – including its diameter, branching pattern, and the distance from its origin to the IMA root – were comparable between the groups. Therefore, another anatomical basis must exist to explain these divergent perfusion patterns. By correlating perfusion outcomes with preoperative CTA findings, we identified distinct anatomical correlates. All seven patients with a prolonged MPD were found to have an AMCA. Conversely, all four patients in the HT group who exhibited a critically short MPD lacked a direct communicating branch between the ascending LCA and the left branch of the MCA. Given these findings, we posit that these divergent responses are more likely attributable to underlying variations in the compensatory capacity of the marginal artery – such as via the Arc of Riolan^[12,21]^ – or possibly related to anatomical variations like marginal artery discontinuity near the splenic flexure^[22]^. This finding underscores the critical need for individualized, intraoperative perfusion assessment and points toward the compensatory capacity of the marginal artery as a crucial area for future investigation.

LSCI findings directly influenced intraoperative decision-making regarding the transection line in 10% of all cases. Notably, these adjustments occurred significantly more frequently in the HT group (16.7% vs. 0% in the LT group), particularly among patients with short MPD, suggesting a higher incidence of suboptimal perfusion with HT. We hypothesize that these LSCI-guided corrections, by ensuring that the anastomosis was constructed from a well-perfused bowel segment of sufficient length to maintain low tension, contributed substantially to the remarkably low overall AL rate of 3.3% observed in our cohort. This rate is favorable when compared to typical outcomes reported with ICG fluorescence guidance, which are 8% in the AVOID trial^[18]^ and 5.2% in the ICG-COLORAL trial^[23]^. While this comparison is encouraging, it must be interpreted with caution due to the inherent differences in study design, patient populations, and potential selection bias between our single-center cohort and these large, multi-center randomized controlled trials. Taken together, our findings suggest that HT may carry a higher intrinsic risk of compromising bowel perfusion and increasing AL risk. However, real-time perfusion assessment with LSCI provides an effective intraoperative safeguard, allowing early identification and correction of suboptimal transection lines prior to anastomosis^[20]^. Our results align with reports like Skinner *et al*, demonstrating that advanced visualization techniques frequently alter surgical plans^[20]^.

Regarding perfusion intensity at the transection line, measured by the SFI, we found no statistically significant difference between the HT and LT groups, although values trended higher in the LT group. This contrasts with findings by Komen *et al*^[24]^, who reported significantly higher flow ratios with LT using laser Doppler flowmetry. This discrepancy might stem from methodological differences: laser Doppler provides point measurements, while LSCI assesses a wider area, and critically, our transection lines were selected within well-perfused segments based on LSCI, whereas Komen *et al* did not explicitly control for this^[24]^. Observing the same favorable AL rate in both groups indicates that while HT may potentially reduce proximal perfusion intensity, it does not necessarily compromise anastomotic safety when the transection is performed within a demonstrably well-perfused region, as guided by LSCI in this study.

### Limitations

This study has several limitations inherent to its design. First, as a single-center pilot study, the generalizability of our findings may be constrained. The outcomes could be influenced by factors specific to our institution, including the surgical team’s expertise, local patient demographics, and established institutional protocols. Second, although propensity score matching was employed to minimize selection bias, the limited sample size restricts the statistical power of our analysis. Finally, the short follow-up period precludes a comprehensive assessment of long-term oncological and functional outcomes. Therefore, to validate these promising preliminary findings and ensure their broader applicability, future large-scale, multi-center studies are essential.

## Conclusion

In conclusion, our results from this pilot study are hypothesis-generating. These findings suggest that the debate between high and low tie may be overshadowed by a more critical factor: the profound inter-patient variability in bowel perfusion. While our data confirm an intrinsic risk of critically compromised perfusion length exclusively within the HT cohort, the central finding is that intraoperative assessment with LSCI can effectively identify these high-risk individuals and guide corrective surgical revisions. By doing so, LSCI acted as a crucial tool to mitigate this intrinsic risk, leading to equally low AL rates across both groups. This work challenges the paradigm of a one-size-fits-all surgical approach, underscoring that anastomotic safety is dictated less by the IMA ligation level itself and more by the individualized. Future large-scale studies are warranted to validate these findings and specifically investigate the role of the marginal artery’s compensatory capacity, paving the way for data-driven, personalized surgical strategies to enhance patient outcomes.

## Data Availability

The data that support the findings of this study are available from the Ningbo Medical Center Lihuili Hospital, but restrictions apply to the availability of these data, which were used under institutional permission for the current study and are therefore not publicly available. However, data may be made available from the corresponding author upon reasonable request and with permission from the Ningbo Medical Center Lihuili Hospital.

## Research registration unique identifying number (UIN)

Name of the registry: ClinicalTrials.gov.Unique identifying number or registration ID: NCT06982664.Hyperlink to the registration: https://clinicaltrials.gov/study/NCT06982664.
